# The Congo red derivative FSB binds to curli amyloid fibers and specifically stains curliated *E*. *coli*

**DOI:** 10.1371/journal.pone.0203226

**Published:** 2018-08-30

**Authors:** Courtney Reichhardt, Lynette Cegelski

**Affiliations:** Department of Chemistry, Stanford University, Stanford, California, United States of America; Universidade Federal do Rio de Janeiro, BRAZIL

## Abstract

The Congo red derivative (*E*,*E)*-1-fluoro-2,5-bis(3-hydroxycarbonyl-4-hydroxy) styrylbenzene (FSB) specifically stains the functional amyloid curli in *Escherichia coli* biofilms. FSB binds to curli with similar affinity as Congo red, yet exhibits much greater fluorescence upon binding to curli as compared to Congo red and does not exhibit undesired binding to the cellulosic component of the biofilm. Thus, FSB presents a powerful tool to identify and visualize curli in *E*. *coli* biofilms and also enables new biophysical investigations of curli.

## Introduction

Bacteria are able to colonize an astonishing range of environments. Across these environments, bacteria tend to associate with surfaces which improves their access to nutritive, protective, or otherwise beneficial environments [1]. To this end, bacteria employ a range of cell-surface fibers, adhesins, and receptors to interact with their environments [2–7]. As one of their adhesive strategies, many bacterial species can produce cell surface-associated amyloid fibers, which have a variety of functions including cell adhesion, biofilm formation, and virulence [2,6–12]. The functional amyloid fibers assembled by *Escherichia coli* are called curli [13]. Curli production is prevalent among uropathogenic *E*. *coli* (UPEC) clinical isolates, the major causative agent of urinary tract infections including acute and chronic and recurrent infections [14]. UPEC bacteria can invade host bladder epithelial cells to assemble biofilm-like intracellular bacterial communities that are able to withstand antibiotic treatment as well as disperse to infect more cells [15]. Curli is the primary component of the extracellular matrix of agar-biofilms formed by the well-studied UPEC clinical isolate called UTI89 [16,17]. Together only two components, curli and phosphoethanolamine-modified cellulose, compose the insoluble UTI89 extracellular matrix in an approximate 6:1 mass ratio [17,18].

As amyloid, curli fibers are protease resistant and bind to Congo red (CR) and other amyloid dyes [19–21]. In general, amyloid fibers are β-sheet rich structures with β strands oriented perpendicular to the fiber axis and can be adopted by many proteins. However, distinct from amyloids that result from protein misfolding or alternative folding pathways, curli are formed through controlled biogenesis that involves the concerted action of several assembly and chaperone proteins [13,22–24]. The classic amyloid dyes CR and thioflavin T are used for histological identification of amyloid-β (Aβ) in plaques of Alzheimer’s disease brain sections [25,26], and these dyes also bind to curli [13,19–21]. As observed in other amyloid systems, CR displays increased fluorescence upon binding to curli as well as birefringence under polarized light [21,27]. Additionally, CR is useful as a purification aid for amyloid and cellulosic materials [20]. Despite the prevalent use of amyloid indicator dyes, the modes of binding of fibers to Congo red and similar dyes remain unclear [28]. Several possible ways that CR could orient itself relative to the fibers have been predicted using a range of computational and spectroscopic approaches [29]. These modes include CR arranged either parallel or perpendicular to the fiber axis or intercalated between the β-sheets [28].

However, CR is not specific to curli (or amyloids in general) and binds to other bacterial extracellular features, including cellulose [20,21]. We sought out to identify curli-specific stains for histological investigations of UPEC infections and for microbiological studies of curli expression relative to other extracellular features [18,30,31]. We recently identified curcumin as an alternate curli-specific dye that does not bind cellulose [21]. However, CR and curcumin both exhibit relatively low fluorescence upon binding to curli[21]. The Congo red derivative (*E*,*E)*-1-fluoro-2,5-bis(3-hydroxycarbonyl-4-hydroxy) styrylbenzene (FSB) was synthesized and introduced for use as a diagnostic stain for Aβ [32]. In studies with Aβ, FSB exhibited excellent amyloid specificity, and the fluoro-substitution resulted in increased fluorescence relative to other analogs [32]. The fluoro-substitution also enabled the use of FSB as a magnetic resonance imaging (MRI) contrast agent to highlight Aβ *in vivo* because ^19^F is a nuclear magnetic resonance (NMR) active nucleus [33].

## Material and methods

### UV-Vis and fluorescence spectroscopy

(*E*,*E)*-1-fluoro-2,5-bis(3-hydroxycarbonyl-4-hydroxy) styrylbenzene (FSB) (Dojindo Laboratories, Lot EU604 and FN606) was added from a 0.1% (w/v) stock solution in DMSO to a solution of 0.5 mg/mL of isolated curli to yield a final dye concentration of 12.5 μM. Native curli fibers were isolated from MC4100 as previously described [17]. Curli was prepared as a 0.5 mg/mL solution by resuspending lyophilized curli to the appropriate final volume. The sample was examined by UV-Vis spectroscopy (Perkin Elmer Lambda 35 UV/Vis Spectrometer). The fluorescence emission spectra were obtained for FSB alone and curli-bound FSB upon excitation at 390 nm (Perkin Elmer LS55 Fluorescence Spectrometer). A 10 nm emission slit and 10 nm excitation slit were used, and the scan speed was 50 nm/min. Reported UV-Vis and fluorescence spectra were background subtracted. Comparative UV-Vis and fluorescence spectra were obtained similarly for curli with CR (excitation at 540 nm), again with a final dye concentration of 12.5 μM and curli concentration of 0.5 mg/mL. In order to not exceed the detection limit of the spectrophotometer, the FSB-curli fluorescence spectrum was acquired as a 400-fold dilution.

### Fluorescence microscopy of agar-grown biofilms

The bacterial strains used in the study are: MC4100, MC4100Δ*csg*, UTI89, and UTI89Δ*csgA*. MC4100 is a laboratory *E*.*coli* strain that produces curli as its only extracellular feature, and MC4100Δ*csg* is the isogenic curli-knockout strain. UTI89 is a commonly studied UPEC strain that produces both curli and phosphoethanolamine-modified cellulose extracellularly, and UTI89Δ*csgA* is the isogenic *csgA*-knockout strain. CsgA is the curli fiber monomeric unit. All four strains were grown on YESCA agar for 60 hours at 26°C. Bacterial cells were harvested using an inoculating loop and spread into 100 μL of 1xPBS on a microscope slide. The bacteria were allowed to air dry on the microscope slide prior to staining with 10 μmol/L of FSB. The dye was allowed to incubate on the cells for 10 minutes before the slides were mounted with Fluoromount G (coverslips Fisher 12-544-B). To acquire fluorescence micrographs of the samples, images of MC4100, MC4100Δ*csg*, UTI89, and UTI89Δ*csgA* were captured using an AxioObserver.Z1 inverted microscope equipped with Filter Set 38 HE, Ex 470/40, Em 525/50, for fluorescence images (Zeiss). Similar images were acquired for bacterial cells stained with CR using the appropriate filter (Filter Set 00, Ex 530–585, Em 615).

### Curli-only FSB depletion assay

For every depletion assay, FSB was added from a 0.1% (w/v) stock in DMSO. To determine binding parameters, two complementary assays were performed, both in 10 mM Tris, 170 mM NaCl, pH 7.4. In the first assay, the FSB concentration was held constant (12.5 μM), and the curli concentration was varied from 0.1 to 1 mg/mL. In the second assay, the curli concentration was held constant (0.1 mg/mL), and the FSB concentration was varied from 5 to 20 μM. During each of the assays, curli was incubated with FSB for 10 minutes with rocking at room temperature before centrifugation (13,000 *g* for 10 minutes) to remove curli and bound FSB. The absorbance of the supernatant, which corresponds to the concentration of free FSB, was measured at 365 nm. Each sample was prepared and assayed in triplicate. A standard curve of the absorbance of FSB at its maximum (365 nm) also was obtained. The ligand binding saturation curve was fit using nonlinear regression to obtain binding parameters, where y = (B_max_•x)/(K_d_+x). The molecular weight of the curli monomeric unit, CsgA, is 13 kDa. All fitting of standard curves and binding curves was performed in software written for Igor Pro (WaveMetrics, Lake Oswego, OR, USA).

### Transmission electron microscopy (TEM) of FSB bound curli

Negative staining transmission electron microscopy (TEM) was performed on curli samples (1 mg/mL) suspended in 10 mM Tris buffer (pH 7.4) with and without incubation with 12.5 μM FSB (15 min, room temperature). Samples were applied to 300-mesh copper grids coated with Formvar film (Electron Microscopy Sciences, Hatfield, PA) for 2 min and then rinsed in deionized water. The samples were negatively stained with 2% uranyl acetate for 90 s, excess stain was wicked off with filter paper, and then the sample was air-dried. Microscopy was performed on the JEM-1400 (JEOL, LLC).

## Results and discussion

### Spectral analysis of FSB binding to curli

**Fig 1:**
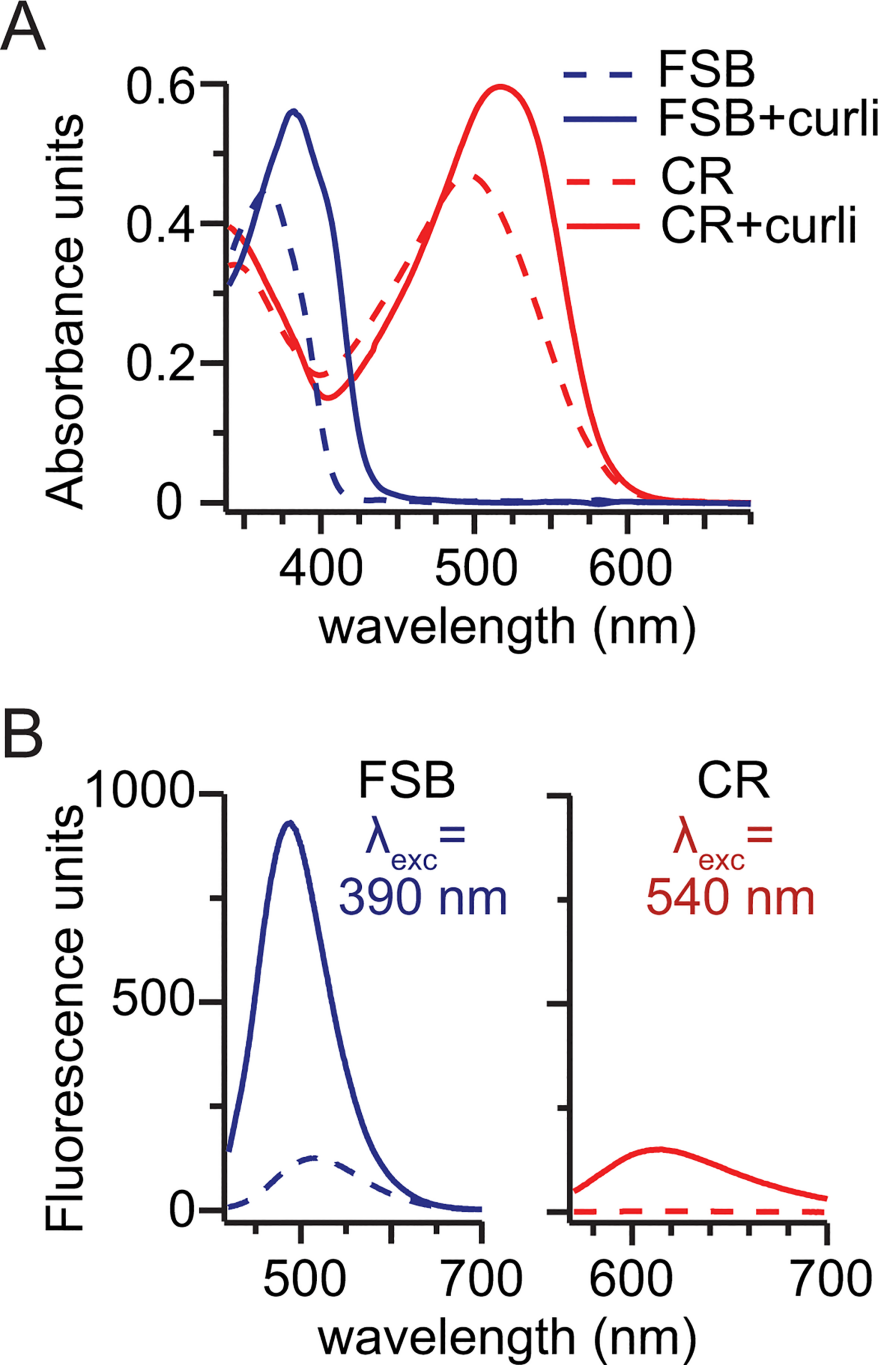
The spectral properties of curli-bound FSB. (A) The UV-Vis spectra indicate that binding of FSB to curli results in a red shift in the absorbance spectrum. Specifically, the absorbance spectrum of curli-bound FSB is red-shifted from a *λ*_max_ of 361 nm to 386 nm (red shift = 25 nm), which is greater than the 10 nm red shift observed for CR bound to curli. (B) The fluorescence emission spectra of each dye alone and in the curli-bound samples were obtained upon excitation at 540 nm for CR and at 390 nm for FSB. FSB exhibits significantly enhanced fluorescence when bound to curli as compared to Congo red. In fact, the curli-bound FSB sample was diluted 400-fold relative to the curli-bound CR sample to prevent exceeding the detection range of the spectrophotometer.

CR exhibits a red-shift in absorbance and fluorescence when bound to amyloid fibers. To determine whether curli-bound FSB would exhibit similar spectral properties, FSB was added to a purified aliquot of curli and the absorbance and fluorescence spectra were collected. The ultraviolet-visible (UV-Vis) and fluorescence spectra of curli-bound FSB were compared to those of curli-bound CR. As shown in Fig 1, the UV-Vis spectrum of curli-bound FSB is red-shifted from a *λ*_max_ of 361 nm to 386 nm (17 nm red shift). This is comparable to the red-shift observed for curli-bound CR (*λ*_max_ of 496 nm to 516 nm; 20 nm red shift). The fluorescence emission spectra of each dye alone and in the curli-bound samples were also obtained upon excitation at 540 nm for CR and at 390 nm for FSB. Especially as compared to CR, FSB exhibits significantly enhanced fluorescence when bound to curli. In fact, to prevent exceeding the detection range of the spectrophotometer, the curli-bound FSB sample had to be diluted 400-fold relative to the curli-bound CR sample. This dramatic increase in fluorescence suggests that FSB is a much more sensitive indicator of curli than CR.

### FSB specificity for curli in agar-biofilm samples

**Fig 2:**
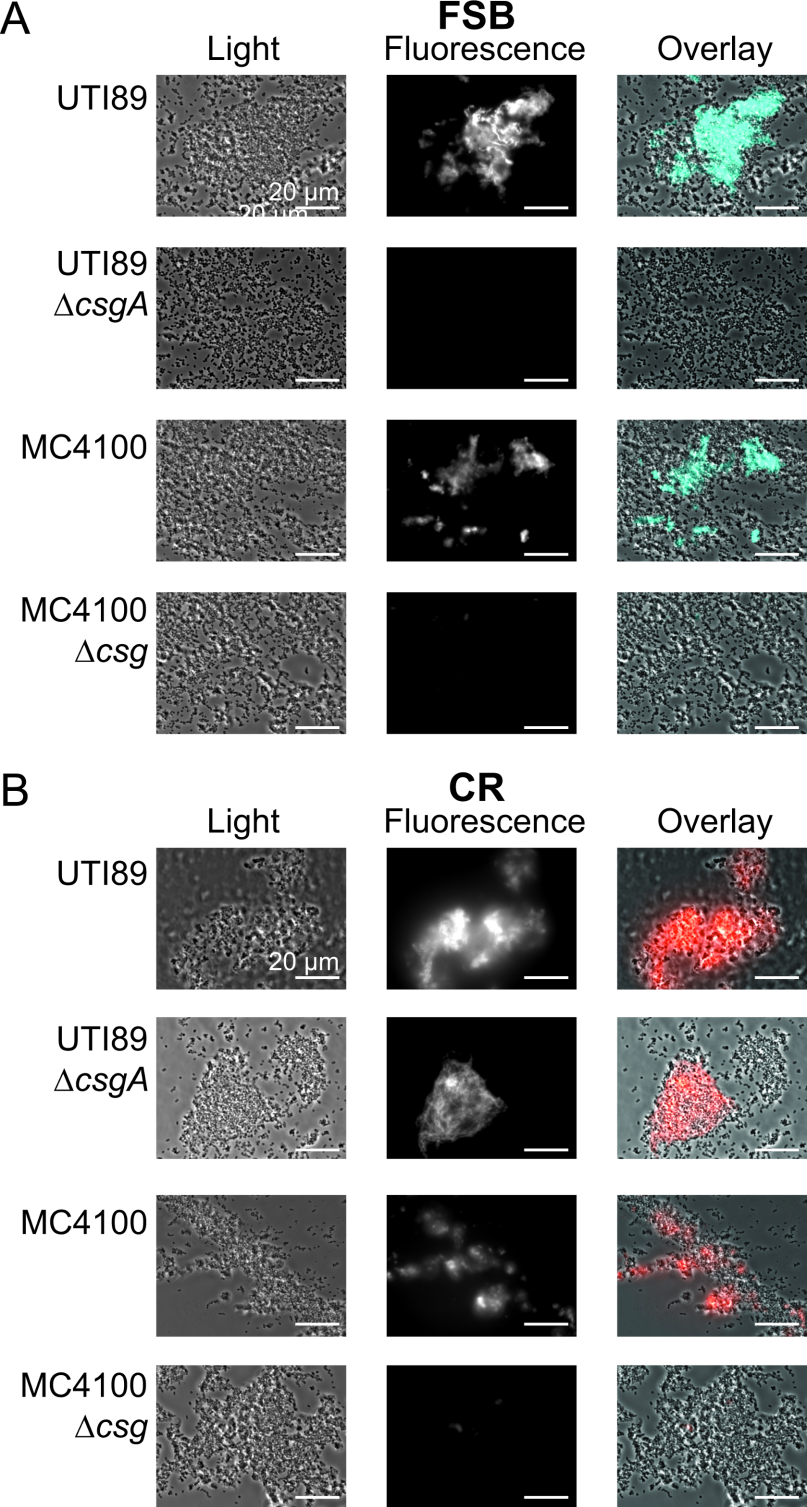
Fluorescence microscopy of *E*. *coli* agar biofilms. Light microscopy images, fluorescence microscopy images, and overlay images of four different strains stained with FSB and CR are shown. The strains studied are: MC4100, MC4100Δ*csg*, UTI89, and UTI89Δ*csgA*. MC4100 is a laboratory *E*.*coli* strain that produces curli as its only extracellular feature, and MC4100Δ*csg* is the isogenic curli-knockout strain. UTI89 is a commonly studied UPEC strain that produces both curli and phosphoethanolamine-modified cellulose extracellularly, and UTI89Δ*csgA* is the isogenic *csgA*-knockout strain. (A) FSB stained only curli-producing bacteria (MC4100 and UTI89). (B) CR also binds to cellulose and is not specific for curli. This is evident as CR stained MC4100 and UTI89 as well as UTI89Δ*csgA*, which only produces extracellular cellulose but not curli. MC4100Δ*csg* does not produce either curli or cellulose, and it was not stained by either FSB or CR. Representative images from three experiments are shown. The scale bars represent 20 μm.

**Fig 3:**
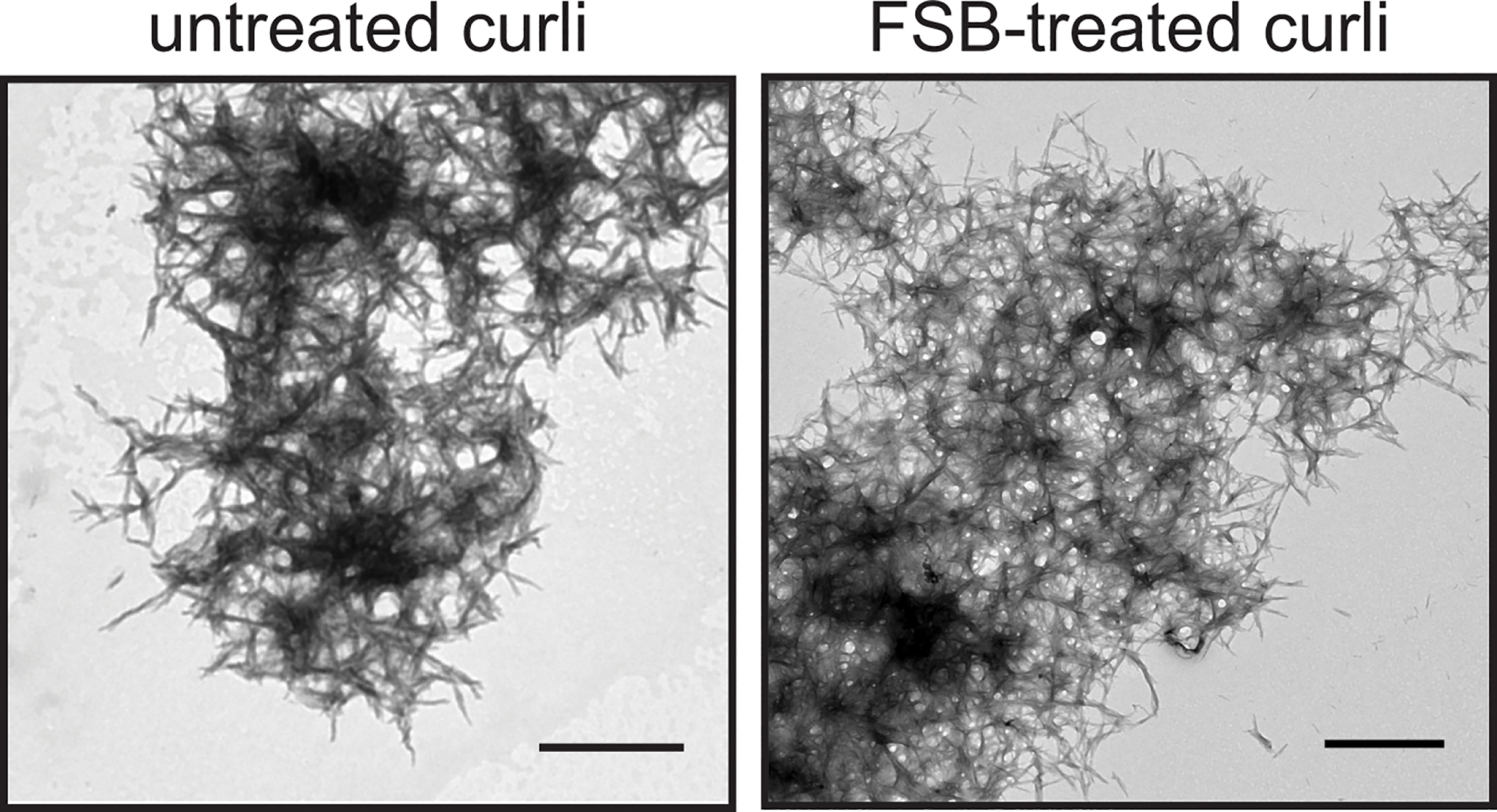
TEM images of curli. Untreated curli are morphologically similar to FSB treated curli. The scale bars represent 1 μm.

The use of dyes with molecular specificity for curli will be valuable in identifying curli within *in vivo* biofilms grown in the laboratory or in the context of samples collected from human infections. Specificity of FSB for curli within bacterial communities was examined by fluorescence microscopy of FSB-stained agar-grown bacteria. Fluorescence microscopy images of MC4100, MC4100Δ*csg*, UTI89, and UTI89Δ*csgA* stained with FSB are shown in Fig 2. Only MC4100 and UTI89 agar biofilms, which produce curli, exhibited fluorescence when stained with FSB. This result is in contrast to that from a similar assay employing CR. As CR binds promiscuously to amyloids and beta-glucans, both curli-producing strains (MC4100 and UTI89) and a non-curliated cellulose-producing strain (UTI89Δ*csgA*) exhibited fluorescence when stained with CR [21]. Interestingly, only a subpopulation of bacteria appeared to stain with either of the dyes, FSB or CR. This suggests that not all of the bacteria are associated with matrix molecules (either curli or cellulose). Whether this is due to differences in matrix production or sample processing would need to be explored further. Thus, FSB appears to bind specifically to curli and not to other extracellular features. Furthermore, transmission electron micrographs of curli samples without FSB treatment are indistinguishable from those of curli with FSB treatment (Fig 3). Together, these results indicate that FSB acts as an indicator dye and does not influence curli morphology.

### Binding affinity of FSB to curli

**Fig 4:**
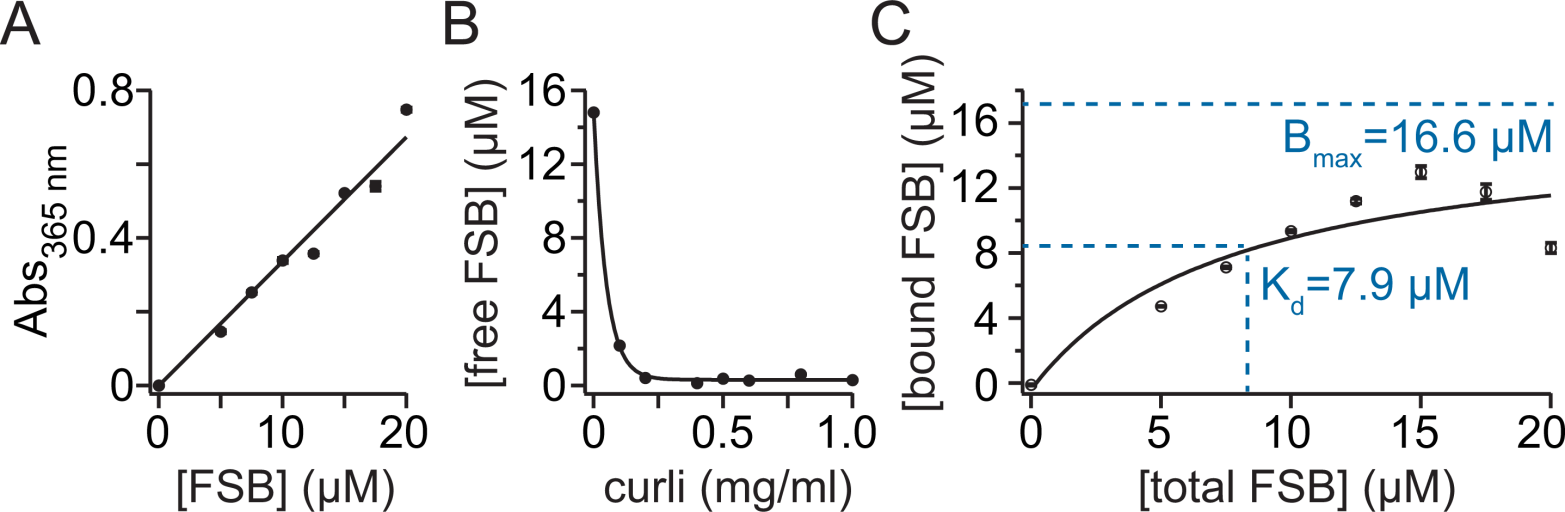
Curli-only FSB binding assay. (A) A standard curve of the FSB absorbance versus concentration was obtained in order to determine the molar absorptivity of FSB at 365 nm (ε_365nm_ = 33.68 mM^-1^cm^-1^; r^2^ = 0.892). (B) Incubation of FSB with increasing amounts of curli resulted in increasing depletion of FSB from solution. (C) A saturation binding curve obtained by varying the FSB concentration. The curve was fit to obtain binding parameters, where y = (B_max_•x)/(K_d_+x).

A curli-FSB binding assay was performed as described previously for curli-CR complexes in order to determine a dissociation constant for FSB binding to native curli fibers isolated from the wild-type curli-producing strain, MC4100 [10]. A standard curve of the FSB absorbance versus concentration was obtained in order to determine the molar absorptivity of FSB at 365 nm (ε_365nm_ = 33.68 mM^-1^cm^-1^) (Fig 4A). To determine the binding parameters, two assays were performed. In the first assay, purified curli was incubated with FSB and centrifuged to remove curli and bound FSB from solution (Fig 4B). Incubation of FSB with increasing amounts of curli resulted in increasing depletion of FSB from solution (Fig 4B). For the second assay, the FSB concentration was then varied in subsequent incubations to obtain a ligand binding saturation curve (Fig 4C). By fitting the curve, the K_d_ was determined to be 7.9 μM, with a B_max_ of 16.6 μM for a curli concentration of 0.1 mg/mL. This K_d_ value is within the range of those of CR binding to curli and other amyloid fibrils (1–10 μM) [19,34]. The number of binding sites for FSB per CsgA, the curli monomeric unit, was estimated using the ratio of [CsgA]/Bmax, where [CsgA] = 7.7 μM, and was found to be approximately two binding sites per CsgA. This estimate assumes even access of FSB to each monomeric unit which may not be true due to interfibril packing.

## Conclusions

The results of this study demonstrate that FSB is useful as a specific and sensitive indicator of curli functional amyloid fibers. The fluorescence of curli-bound FSB is much greater than for curli-bound CR. Specifically, the fluorescence intensity of a 400-fold dilution of curli with FSB is more than six times greater than the fluorescence intensity of a corresponding undiluted solution of curli with CR. Furthermore, FSB is highly specific for curli and, thus, superior to CR for staining of relevant biofilm samples with detection by fluorescence microscopy. FSB may be useful as a selective stain for curli in histological samples and for investigating localization of curli relative to other biofilm matrix components in fluorescence microscopy experiments, including confocal scanning laser microscopy. Additionally, the fluoro-substitution of FSB makes the dye useful for ^19^F NMR studies to determine the binding mode of curli—and possibly other amyloid fibers—to FSB, a CR analog.
